# Should de Winter T-Wave Electrocardiography Pattern Be Treated as ST-Segment Elevation Myocardial Infarction Equivalent with Consequent Reperfusion? A Dilemmatic Experience in Rural Area of Indonesia

**DOI:** 10.1155/2018/6868204

**Published:** 2018-03-31

**Authors:** Raymond Pranata, Ian Huang, Vito Damay

**Affiliations:** ^1^Faculty of Medicine, Universitas Pelita Harapan, Tangerang, Banten, Indonesia; ^2^Tabanan General Hospital, Tabanan, Bali, Indonesia; ^3^Siloam Hospitals Lippo Village, Tangerang, Banten, Indonesia

## Abstract

**Background:**

Although de Winter T-wave electrocardiography pattern is rare, it signifies proximal left anterior descending artery occlusion and is often unrecognized by physicians. The aim of this case report was to highlight the dilemma in the management of a patient with de Winter T-wave pattern in the hospital without interventional cardiology facility.

**Case Presentation:**

A 65-year-old male presented with typical chest pain since 2 hours before admission, and ECG showed sinus rhythm of 57 bpm and >1 mm upsloping ST depression with symmetric tall T in lead V_2-3_ characteristic of de Winter T-wave ECG pattern. He was given dual antiplatelet therapy, nitrate, statin, and anticoagulant. He refused referral to interventional cardiology available hospital. 3 hours after admission, the electrocardiography transformed into Q-waves consistent with final stages of acute STEMI and ST-segment elevation that barely meets the threshold in the guideline, and thrombolytic was administered and successful. There is a suggestion that de Winter T-wave electrocardiography should be treated as ST-segment myocardial infarction equivalent and should undergo coronary angiography; however, not every hospital has the luxury of interventional cardiology facility. The other modality for reperfusion is thrombolysis; however, without a clear guideline and scarcity of study, we prefer to resort to conservative treatment. “Fortunately,” transformation into ST-segment elevation helps us to determine the course of action which is reperfusion using thrombolytic.

**Conclusions:**

de Winter T-wave ECG pattern is not mentioned in any guidelines regarding acute coronary syndromes, and there are no clear recommendations. Physicians in rural area without interventional cardiology facility face a dilemma with the lack of evidence-based guideline. Fibrinolytic may be appropriate in those without contraindications with strong chest pain consistent with acute coronary occlusion, less than 3 hours of symptoms, and convincing de Winter T-wave ECG pattern for a rural non-PCI hospital far away from PCI capable hospital.

## 1. Background

In 2008, de Winter et al. reported an electrocardiography (ECG) pattern that is found in about 2% of patients with proximal left anterior descending artery occlusion (acute anterior myocardial infarction) and is often unrecognized by physicians [1, 2]. Although de Winter T-wave ECG pattern was suggested to be managed as STEMI equivalent [3], it was not mentioned in recent guidelines by the European Heart Society (ESC) and the American Heart Association (AHA) [4, 5]. Hence, a question arises regarding whether or not this pattern be treated with fibrinolytic in an area where cardiac catheter laboratory (cath lab) is not readily available. The aim of this case report was to highlight the dilemma in the management of a patient with de Winter T-wave pattern in hospital without interventional cardiology facility (non-PCI hospital).

## 2. Case Presentation

**Figure 1:**
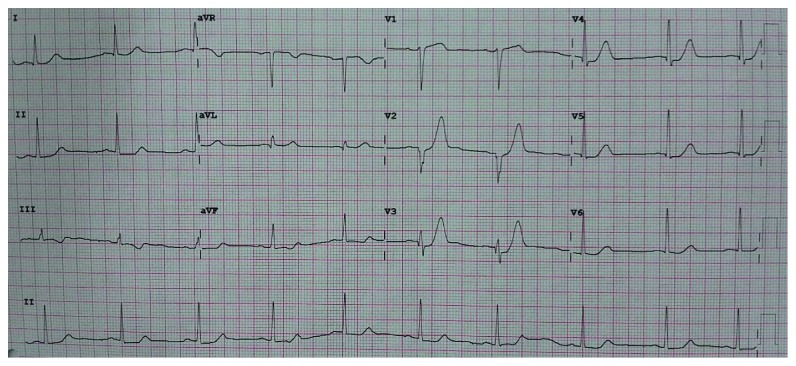
Initial ECG showing >1 mm upsloping ST depression with symmetric tall T in lead V_2–6_ characteristic of de Winter ECG pattern.

**Figure 2:**
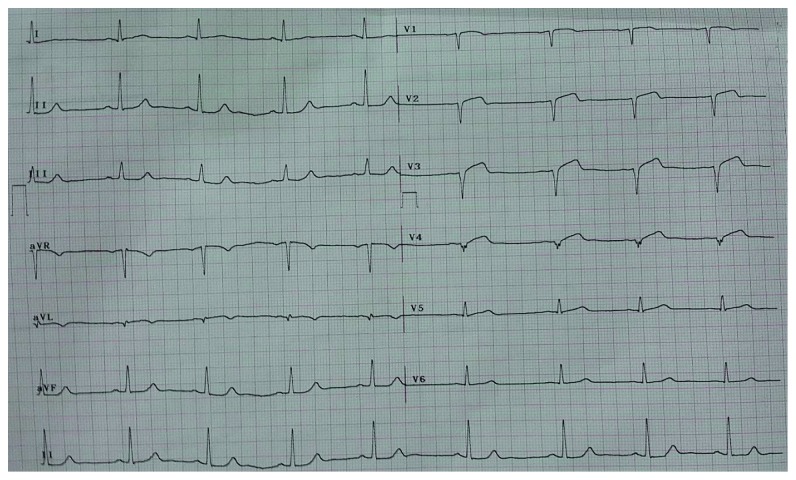
ECG showing progression into ST-segment elevation myocardial infarction.

**Figure 3:**
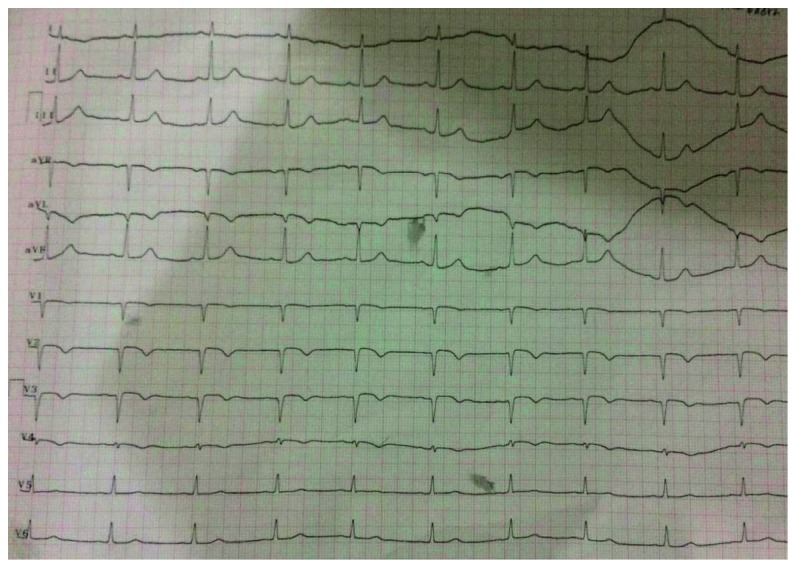
ECG after fibrinolysis showing resolution of ST-segment elevation.

A 65-year-old male presented to the emergency department with typical chest pain at rest since 2 hours before admission after eating. The patient denied shortness of breath and previous episode of chest pain before admission. He had a past medical history of poorly controlled hypertension and was taking captopril. History of diabetes, smoking, and previous stroke or myocardial infarction was denied. On physical examination, blood pressure was 150/90 mmHg, heart rate was 57x/minute, and respiratory rate was 20x/minute, and the cardiopulmonary examination was within normal limits. ECG showed sinus rhythm of 57 bpm and >1 mm upsloping ST depression with symmetric tall T in lead V_2-3_ characteristic of de Winter T-wave ECG pattern (Figure 1). Routine blood examination, AST/ALT, BUN, and serum creatinine were within normal limits. Sodium was 137 mmol/L, and potassium was 3.4 mmol/L denoting that tall T was not due to hyperkalemia. Troponin at the 2nd hour after onset was negative. The patient was diagnosed with acute coronary syndrome with suspected de Winter T-wave ECG pattern. Aspirin 160 mg, clopidogrel 300 mg, sublingual isosorbide dinitrate, simvastatin 20 mg (atorvastatin was not available), and enoxaparin 0.4 mg subcutaneous were administered. The patient refused referral to the interventional cardiology capable hospital due to distance and socioeconomic causes. Repeat ECG was done 3 hours later showing Q-waves developing in V_2–4_, consistent with the final stages of acute STEMI accompanied with ST-segment elevation in lead V_2–4_ which barely meets the 2 mm threshold for those precordial leads as per the guidelines (Figure 2). Elevated troponin at the 5th hour confirmed the presence of myocardial infarction. The patient still experienced sustained and severe chest pain although not as intense as initial presentation. Streptokinase (1.5 million units) was administered, and chest pain subsided with a resolution of ST-segment elevation (Figure 3). The patient was discharged on the 5th day after admission.

## 3. Discussion

The de Winter T-wave ECG pattern may signify proximal left anterior descending artery occlusion and was suggested as ST-segment elevation myocardial infarction (STEMI) equivalent [1–3]. The de Winter T-wave ECG pattern was also shown to have positive predictive values of 95.2% (95% confidence interval: 76.2–99.9%), 100% (69.2–100.0%), and 100% (51.7–100%) for acute coronary occlusions in the three diagnostic studies [6]. Verouden et al. also reported that approximately 50% of those with de Winter T-wave ECG pattern had a wraparound LAD which is associated with larger area of ischemia [2]. However, despite these findings, de Winter T-wave ECG pattern is yet to be included in ESC guidelines for management of acute coronary syndromes in both persistent (2017) or without persistent ST-elevation (2015) and ACC/AHA guidelines regarding STEMI (2013) and NSTEMI (2014) [4, 5].

Ideally, the presence of de Winter T-wave ECG should be treated as urgent as STEMI with catheter lab activation for coronary angiography and possible stenting. This was not possible in our case due to a limited facility in the region [3]. Thrombolysis was initially avoided because de Winter T-wave ECG is currently not an indication for fibrinolysis even in latest guidelines, and there was no clear-cut evidence of acute coronary occlusion. The lack of the evidence-based guidelines for the aforementioned condition compelled us to choose dual antiplatelet therapy and anticoagulant for the initial mainstay of therapy. Evolution from suspected de Winter T-wave pattern to the final stage of acute STEMI was observed three hours after admission. This helped us in ushering the direction of management, as the guideline for STEMI is straightforward in terms of reperfusion. Thrombolysis was initiated, and repeat ECG showed resolution of chest pain and elevated ST-Segment which proves the acute coronary occlusion (anterior myocardial infarction).

Whether de Winter T-wave ECG pattern can evolve into STEMI is debatable, and there are arguments that de Winter T-wave pattern is a part of STEMI evolution which was impeded with aggressive antithrombotic/antiplatelet therapy [7]. Another opinion states that ECG evolution into STEMI was actually indicative of hyperacute T-wave instead of de Winter T-wave ECG pattern, in which the latter usually progresses directly to sign of transmural infarction on ECG [3, 8]. A more diplomatic view was the categorization of de Winter T-wave ECG pattern into those 2 debated groups, respectively [9]. The presence of Q-waves in our patient with ST-segment elevation which barely meets 2 mm suggests transformation into final stages of acute STEMI; hence, our patient showed both evolution into STEMI and signs of transmural infarction (Q-wave).

Regardless of the debate, the most important issue is to recognize this ECG pattern and prevent the delay in management. Delay leads to a higher total ischemic time which is related to higher mortality in STEMI; however, whether the same applies to de Winter T-wave ECG is unclear [10].

## 4. Conclusions

The de Winter T-wave ECG pattern is not mentioned in any guidelines regarding acute coronary syndromes, and there are no clear recommendations. Physicians in rural area without interventional cardiology facility face a dilemma with the lack of evidence-based guideline and prefer to resort to a conservative strategy rather than potentially doing harm. Such approach may or may not lead to the best possible outcome but is the only choice in the middle of scarcity. Fibrinolysis may be considered in a young patient (without usual contraindications) who arrives with strong chest pain consistent with acute coronary occlusion, less than 3 hours of symptoms, and with convincing de Winter T-wave ECG pattern (especially if a prior baseline ECG is available for comparison) for a rural non-PCI hospital far away from PCI capable hospital. Discussion with the interventional cardiologist at the PCI hospital whenever possible should also be done in these challenging cases to optimize real-time decision-making.

## Abbreviations

AST: Aspartate aminotransferase
ALT: Alanine aminotransferase
BUN: Blood urea nitrogen
ECG: Electrocardiogram
STEMI: ST-segment elevation myocardial infarction
NSTEMI: Non-ST-segment elevation myocardial infarction.
